# Annotations

**Published:** 1899-03-04

**Authors:** 


### Annotations.

### The Saprophytic Life of Pathogenic Bacteria.

In the very interesting Milroy Lectures now being delivered at the Royal College of Physicians, Dr. Poore describes the action of the earth in relation to the preservation and destruction of contagia, pointing out that the chemical composition of earth is constantly changing, and that its fitness for supporting the life of the various micro-organisms that are found in it varies accordingly. Each of these microbes presumably grows best in what one may call its optimum conditions, and the smallest alteration in these conditions may turn the scale and decide which among the competing organisms shall obtain the mastery. In illustration of the curious exactitude of the conditions without which certain fungi will not grow, and of their extraordinary development whenever these conditions are fulfilled, Dr. Poore refers to the growth of mushrooms. "Some of the larger saprophytes," he says, "such, for instance, as the common mushroom, require no little skill for their artificial production, involving far more attention to exact details than is necessary with the ordinary green-leaved garden plants. We know that for a few weeks in the autumn they may appear in great numbers in dry pastures where horses have been fed, provided that the conditions of the air as to temperature, light, and moisture be favourable, and we also know that directly the necessary conditions fail the mushroom harvest is at an end." And it is clear that "the optimum conditions for the growth and development of these shortlived and delicate organisms must be marvellously subtle, probably quite beyond the ken of the chemist." Referring then to certain diseases, such, for example, as anthrax and tetanus, the germs of which are known to grow freely in certain soils, attention is drawn to the rarity with which men are attacked by them even when working on land which has almost certainly been infected. We are thus brought face to face with the importance of the extra-corporeal phases of microbic life in the maintenance of continuity of infection. Unfortunately, we seem to know but little in regard to the exact conditions under which the several pathogenic organisms are best able to maintain their existence in saprophytic form. Something we know about the bacillus of tuberculosis, and something we are beginning to know about that of enteric fever, but much more knowledge in this direction is badly wanted. It is true enough, no doubt, as has been well said, that "the proper study of mankind is man," but this is just what the pathologist must unlearn if he would get to the bottom of the etiology, and especially of the epidemiology, of infectious ailments. No longer must he be content to study the action of the microbe within man's frame or the methods by which it brews toxins and sets up disease, but he must trace its life history outside, and find out what these infectious particles are doing and how they are living through the long interval that sometimes elapses between the termination of one "case" and the development of another. One fine morning we look out upon our paddock and find it full of mushrooms; and another, although we know that the spore is present just the same, there is not a mushroom to be seen It was the conditions, not the seed alone, that made them grow. And this is a parable.

### Incipient Insanity.

It is somewhat of a scandal that such difficulties should be placed in the way of the early treatment of insanity as exist in the present state of the law in England. In few other diseases is it so important that the very earliest symptoms should receive attention, and in few is it so necessary that the patient should be removed from amid the surroundings of his ordinary life and placed under the care of someone skilled in the management of such cases. Yet directly one tries to do the best that can be done for a patient suffering from nervous breakdown and from those warning symptoms which, if untreated, are known often to end in actual insanity, one finds oneself hampered on every side by legal restrictions. These restrictions have been carefully arranged, and with the best possible intention, their object being to prevent the possibility of a sane person being imprisoned on the plea of lunacy, but they have unfortunately had the effect of leaving at large a crowd of incipient lunatics, sometimes wandering about the streets, potential murderers, liable to be led into any crime on the slightest provocation; sometimes ostensibly attending to their business, but really scattering their substance, and ruining their families under insane delusions, and yet, by virtue of the existing law, incapable of detention or restraint; waiting, always waiting, for such symptoms to develop as shall make it safe for a medical practitioner to give a certificate of insanity, What is wanted is some arrangement by which it shall be rendered possible to deal as freely with diseases affecting the thinking portions of the brain as we now do, in hospitals and other institutions, with those affecting its other functions; and, for this purpose, all that seems necessary is to assimilate the English to the Scottish law so far as regards the treatment of cases in which insanity is not yet confirmed. We

ANNOTATIONS—*continued.*

regard, therefore, with full approval, although perhaps not with such great hopefulness as some do, the proposals made by the joint committee of the British Medical and the Medico-Psychological Associations, which has lately submitted to the Lord Chancellor a draft clause providing for the temporary care of persons suffering from incipient unconfirmed mental disease. According to this proposal a person certified by a medical practitioner to be suffering from mental disease which is not confirmed might be placed under proper care in a private home for a period not exceeding six months, due notice being, of course, given to the Commissioners in Lunacy, who would have a right to visit the patient. In this way all the safety to the public which is imagined to spring from the supervision of the Commissioners in Lunacy would be secured to the "borderland" cases; and yet such cases could be placed under care and supervision at the earliest possible moment, long before the malady had become so pronounced as to justify the friends, the medical men, and the magistrate in formally committing the patient to an asylum.

### Cottage Homes for the Aged Poor.

Whatever may be the fate of the Bill which Mr. Hutton has introduced into the House of Commons for establishing cottage homes for the aged poor who would otherwise have to go to the workhouse, it shows the growing feeling in the country to discriminate between those whose poverty is due to misfortune and those who are the main cause of their own distress. To settle who is fit for the workhouse and who for the cottage, with its greater comfort and higher social consideration, may prove a difficult matter, and there is always a danger that favouritism and influence may enter in; but Mr. Hutton has introduced one suggestion that may help to fix a claim. He desires to extend the benefit of these cottages beyond the absolute pauper who has nothing, to the man who has struggled to save enough for his old age. Nothing is more sad than to see old people compelled to spend all the little savings of a lifetime, gathered together by such abstinence as most people who save have no conception of, and, at the end of all, forced to enter the hated workhouse which it was the aim of this severe economy to avoid. The people who have shown thrift and independence and self-respect are the most to be pitied when at last they sink to dependence, and anything that would eke out their humble savings, instead of forcing them to spend them and then come to pauperism, is a thing to be devoutly sought after. This Mr. Hutton's Bill seems to provide, or would provide, if it could be made clear to the world that those who occupied these cottage homes were not, in the ordinary sense of the word, paupers. Now, to this end, would it not be well to limit admission to these homes—at least in the first place—to those who had shown by the possession of some savings that they were industrious, thrifty, and willing to help themselves? This limitation would at once put the occupant of the cottage on a different platform from the ordinary pauper; and while it would put the respectable poor in a better position than as a rule they can now hope for, it would justify guardians in making the workhouse a less attractive place than at present, with the inmates so mixed, the heart of the average man can desire it to be. There are also cases where the old people themselves are destitute, but have children who can do something for them, though, perhaps, they cannot maintain them wholly. But if accommodation in a cottage could be furnished, they might be able to provide enough for their other wants. It seems to us that the possession of private means, however small, should form a preferential claim to admission into this superior home, which yet is provided out of the public funds. It is doubtful if any objection would be raised to this; for almost everybody is willing to give charity, but is continually hampered by the thought that the donations are as likely to do harm as good, by sapping the moral fibre and self helpfulness of the recipient. To make the possession of savings a preferential claim, even if not a *sine qua non*, to entrance into a cottage home, would at once elevate their occupants from the rank of paupers, and encourage those among the poor who would show more thrift if they were not now deterred by the thought that the little they can save, even by the severest economy, is not enough to maintain them in their old age, and will only postpone by a year or two the dreaded day when they must enter the workhouse.

### Scarlet Fever and Diphtheria.

For several years back Mr. Shirley Murphy, in his annual reports on the health of London, has shown that both in regard to scarlet fever and diphtheria the period in which the disease is the most prevalent is by no means that in which it is of the severest type as judged of by its case mortality. Now, it is open to the ingenious speculator to frame for himself various hypotheses as to the meaning of this fact. It might be suggested that two types of these diseases run concurrently, and that while one type is highly dangerous, the other, although milder, is far more infectious, possibly in consequence of being less perfectly isolated; or it might be thought that the climatic influences which characterise one part of the year tend to the spread of the disease, while those of another season tend towards its intensification. This year, however, Mr. Murphy has extended his statistical investigations in another direction, and has shown that the seasonal variation of these diseases, although affecting all ages in the same sense, affects them in very different degree. Roughly speaking, it would seem that in the months of greatest prevalence more than the average proportion of sufferers are above five years of age, while in the months of greatest fatality more than the average proportion are below five years old; and, when we bear in mind how enormously greater is the case mortality during the first few years of life than it is at later periods, it is not difficult to understand the greater case mortality of the disease at those seasons of the year when a larger proportion of the patients than usual are at the most dangerous age—that is, under five years old. The question, therefore, now takes another form. It is no longer, Why is the fatality greatest at the time when the general prevalence is least? but, Why do those seasonal influences, whatever they may be, which cause the disease to be so much more prevalent between June and November than at other times of the year affect the older children more than the younger ones?

